# Image aesthetic quality assessment: A method based on deep convolutional capsule network

**DOI:** 10.1371/journal.pone.0331897

**Published:** 2025-09-22

**Authors:** Yuchen Hu, Wu Dong, Yan Zhang, Likun Lu

**Affiliations:** Beijing Institute of Graphic Communication/College of Information Engineering, No. 1 Xinghua Avenue (Band Two), Daxing, Beijing, China; Universita degli Studi di Milano, ITALY

## Abstract

Image aesthetics assessment (IAA) has become a hot research area in recent years due to its extensive application potential. However, existing IAA methods often overlook the importance of spatial information in evaluating image aesthetics. To address this limitation, this study proposes a novel method called the Deep Convolutional Capsule Network (DCCN), which integrates an improved Inception module with a capsule routing mechanism to enhance the representation of spatial features—an essential yet frequently underexplored aspect in aesthetic evaluation. This design enables the model to effectively extract both global and local aesthetic features while maintaining spatial relationships. To the best of our knowledge, this is the first attempt to apply capsule networks in the IAA domain. Experiments conducted on two benchmark datasets, CUHK-PQ and AVA, demonstrate the effectiveness of the proposed method. The DCCN achieves a classification accuracy of 94.79% on CUHK-PQ, and on AVA, it obtains a Pearson Linear Correlation Coefficient (PLCC) of 0.8408 and a Spearman Rank-Ordered Correlation Coefficient (SROCC) of 0.7394. While the DCCN shows promising results, it exhibits sensitivity to style variations and resolution changes and has relatively high inference complexity due to dynamic routing, which may affect deployment in real-time applications.

## Introduction

**Fig 1:**
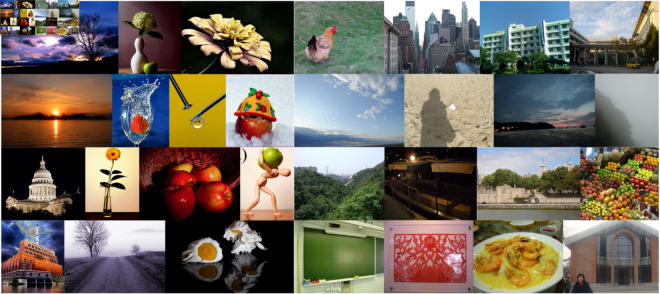
Typical examples of high and low aesthetic quality images. (a) High aesthetic quality images. (b) Low aesthetic quality images.

With the rapid development of Internet technology and the widespread use of smartphones and digital cameras, capturing and sharing images has become remarkably convenient, resulting in an explosive growth of digital images. However, the aesthetic quality of these images varies significantly. Consequently, selecting high-aesthetic-quality images from massive image collections has emerged as a crucial research topic in the fields of image processing and computer vision [1]. Image Aesthetic Assessment (IAA) seeks to computationally model human aesthetic preferences by predicting overall aesthetic scores and attribute-based ratings, reflecting the inherently subjective nature of visual aesthetics [2]. Fig 1 presents representative examples of images with high and low aesthetic quality. On social media platforms, high-aesthetic-quality images are more likely to attract user attention and receive more likes and shares. In industries such as advertising and media publishing, such images can significantly enhance user experience and optimize advertising performance. In the field of artistic design, aesthetically pleasing images can improve the visual appeal and artistic value of creative works.For individual users, posting high-aesthetic-quality images helps establish a positive personal image and increases influence and recognition within social networks. Therefore, in-depth research on image aesthetic quality assessment is of great theoretical significance and holds broad application prospects. It can be applied to improve content quality, optimize recommendation systems, and enhance users’ photographic and editing skills [3].

Aesthetic quality is fundamentally different from general image quality. Traditional image quality assessment (IQA) focuses on detecting degradations such as noise, blur, or compression artifacts, aiming to measure objective visual fidelity [4]. In contrast, IAA deals with highly subjective perceptions, encompassing emotional responses, compositional preferences, and stylistic appreciation [5]. While IQA emphasizes technical correctness, IAA emphasizes visual appeal and personal preference. Therefore, aesthetic assessment presents more nuanced challenges and requires models to capture complex, abstract, and subjective features beyond simple distortions.

In recent years, some research has explored aesthetic assessment from multiple perspectives. For example, Li et al. [6] proposed an attribute-assisted multimodal memory network to integrate visual and textual cues for aesthetic prediction. Soydaner and Wagemans [2] presented a multi-task convolutional neural network that simultaneously predicts overall aesthetic scores and aesthetic attributes. Pan et al. [7] designed an adversarial learning framework to enhance attribute representation for aesthetic assessment. These works highlight the importance of attributes, semantics, and multi-task learning in aesthetic modeling. Although our method focuses on overall aesthetic score prediction, it provides a flexible foundation and can be further extended to attribute-aware and multimodal settings in future research.

In previous studies, researchers have employed handcrafted methods to extract aesthetic features from images. This category of image aesthetic quality assessment algorithms focuses on extracting predefined features that are constructed based on human visual perception and aesthetic theory. The core idea of handcrafted-feature-based methods is to evaluate image aesthetic quality using visual elements such as composition, color, and texture.For instance, Datta et al. utilized 56 features including texture, color, rule of thirds, and region contrast to represent image aesthetics [8]; Ke et al. employed image clarity, contrast, color, and average brightness as aesthetic features [9]; Tong et al. used three features—contrast, vividness, and saliency—to distinguish aesthetic quality [10]; Marchesotti et al. adopted Scale-Invariant Feature Transform (SIFT) and content-based local image descriptors to classify aesthetic quality [11]. Handcrafted-feature-based approaches are generally built upon explicit aesthetic theories, providing relatively strong interpretability for the assessment results. However, these methods suffer from two main limitations. First, handcrafted feature extraction heavily relies on specific aesthetic theories, requiring researchers to possess substantial domain knowledge. Moreover, the aesthetic features considered are often not comprehensive enough to accommodate diverse aesthetic standards. Second, for complex aesthetic attributes such as emotional expression, it is difficult to extract effective features using handcrafted methods.

In contrast, deep learning models are capable of extracting comprehensive aesthetic features from images, thus overcoming the limitations of traditional algorithms. As a result, many recent studies have adopted deep learning-based approaches. In general-purpose image aesthetic quality assessment algorithms based on deep learning, researchers utilize deep learning techniques to automatically extract aesthetic features from images. These models can learn complex aesthetic representations directly from large-scale annotated datasets. Compared with handcrafted-feature-based methods, deep learning approaches can capture more abstract and subtle aesthetic differences, thereby achieving higher accuracy in image aesthetic quality assessment tasks.For instance, Tian et al. proposed a dual-path deep convolutional neural network model [12]; Kao et al. introduced a multi-task deep learning framework [13]; Lu et al. developed a deep multi-patch aggregation network [14]; Wang et al. presented a multi-scene deep learning model [15]; Zhang et al. proposed a multimodal self-and-collaborative attention network that models the relationship between image and textual features [16]; Liu et al. introduced an attention mechanism-based model with holistic nested edge detection, focusing on local and edge features of images [17]; Yang et al. proposed an aesthetic quality assessment model based on color composition and spatial formation, evaluating the aesthetic level by analyzing image color and spatial structure [18]; Li et al. designed a multi-task self-supervised model guided by photographic knowledge [2]; Celona et al. presented a model combining image semantics, artistic style, and composition attributes [19]; Pfister et al. proposed a self-supervised learning method for aesthetic feature representation [20]; Yan et al. developed a semantic-aware multi-task convolutional neural network [21]; and Chen et al. introduced a multi-task network based on scene, aim, and emotion information [22].

In the earlier development of deep learning algorithms, researchers commonly utilized Convolutional Neural Networks (CNNs) to process image data for aesthetic quality assessment. CNNs can automatically extract key features from raw data through multiple layers of convolution, capturing low-level features such as edges and textures, and progressively combining them into more complex high-level features, thereby deepening the understanding of visual patterns [23]. However, CNNs often suffer from significant loss of spatial information due to pooling operations [24]. Although these operations are effective for dimensionality reduction and feature abstraction, they inadvertently strip away spatial details from image data. This loss of fine-grained spatial information is particularly detrimental in tasks that rely on precise element arrangement, such as image aesthetics assessment, where spatial composition plays a crucial role [25].

The spatial information of an image is a critical aspect of image analysis, as it involves the relative positions and layout of elements within the image, directly influencing human visual perception and aesthetic experience [26]. One fundamental principle is balance, which refers to the even distribution of elements within the spatial layout of an image. When the elements in an image are visually distributed evenly or symmetrically, they produce a sense of stability, harmony, and visual pleasure. Another key principle is emphasis and focus. By controlling the relative size, position, and contrast of image elements, viewers’ visual attention and focus can be guided. When important elements are placed in prominent spatial positions within an image, such a layout can draw the viewer’s gaze and convey important information [27]. Proper use of focus helps viewers better understand the theme or narrative conveyed by an image. Therefore, spatial information is of great importance in image aesthetic assessment.

To address the aforementioned limitations of existing CNN- and Transformer-based methods in preserving spatial hierarchies, this study introduces the Capsule Network (CapsNet) [28]. While various CNN and Transformer-based models have been used for IAA, they often struggle to maintain fine-grained spatial relationships that are crucial to human aesthetic perception. Capsule networks, on the other hand, are particularly suited for such tasks due to their inherent ability to preserve spatial hierarchies and semantic entity relationships via dynamic routing. Specifically, it encodes object pose parameters (e.g., position and orientation) into capsule vectors, which are routed via a dynamic routing mechanism to preserve spatial relationships between features [29]. This mechanism avoids the excessive information loss often caused by pooling operations in CNNs and provides a more interpretable representation through vector outputs [30]. Furthermore, to enhance aesthetic feature extraction, we design an improved Inception module without max-pooling and with adjusted kernel sizes to capture multi-scale features while maintaining spatial integrity [31]. These components are integrated into the proposed Deep Convolutional Capsule Network (DCCN), which is designed to comprehensively model spatial structure and aesthetic semantics.

DCCN is designed to comprehensively extract image aesthetic features while preserving spatial information. The major contributions of this study are as follows:

1. The first use of CapsNet in the image aesthetics assessment method is proposed.
2. An improved inception module that enhances aesthetic feature extraction is presented.
3. High accuracy was attained by the DCCN in the binary classification and distribution histogram prediction tasks, as reflected by the notable PLCC and SROCC scores.

## Related work

Methods for aesthetic assessment of images can be categorized into two groups, methods for extracting aesthetic features manually and methods for extracting aesthetic features of images using deep learning methods.

### Manual features

The first category uses manual features. Datta et al. [8] utilized 56 features, including light, colorfulness, saturation, hue, rule of thirds, familiarity measure, texture, size, aspect ratio, region composition, low-depth-of-field indicators, shape convexity and so on, to represent the aesthetic quality of an image. Ke et al. [9] considered six features in their aesthetics assessment approach: spatial distribution of edges, color distribution, hue count, blur, contrast, and average brightness. Tong et al. [10], four features were used to differentiate images with high- and low- aesthetic quality, and these are blurriness, contrast, colorfulness, and saliency. Marchesotti et al. [11] employed generic content-based local image signatures to classify the aesthetic quality of an image. In manual feature-based methods, researchers must acquire extensive knowledge about photographic aesthetics, making it challenging to extract the overall aesthetic features of images.

### Deep learning feature

The second category uses aesthetic features extracted using deep-learning methods. Tian et al. [12] utilized powerful deep convolutional neural networks, whereas Kao et al. [13] developed a multi-task deep learning framework. Lu [14] introduced a double-column deep convolutional neural network, and Wang et al. [15] created a multi-scene deep learning model. Zhang et al. [16] focused on image-text feature relationships with their multimodal self-and-collaborative attention network. Yang et al. [18] assessed image aesthetics using a model that analyzed color composition and spatial formation. Celona et al. [19] combined image semantics, artistic styles, and composition into the aesthetic assessment model. Pfister et al. [20] leveraged a self-attention mechanism for learning aesthetic features, and Yan et al. [21] integrated semantic awareness into a multi-task convolutional neural net-work. However, during the feature extraction process of these deep learning-based methods, spatial information is often lost, potentially overlooking its influence on image aesthetic quality.

## Method

Based on the information provided in Table 1, it is apparent that the methods listed do not explicitly consider spatial location information or incorporate it to a limited extent. For instance, the manual features described by Datta et al. [8], Ke et al. [9], and Tong et al. [10] focused on various aesthetic attributes, such as light, colorfulness, saturation, and contrast, but there was no explicit mention of the spatial location within the image. Similarly, Marchesotti et al. [11] mentioned the use of “generic content-based local image signatures,” which may imply some level of spatial awareness, but do not clearly indicate the extent to which spatial location is factored into their analysis.

**Table 1:** The typical methods of image aesthetic assessment.

Category	Method	Features
**Manual features**	Datta et al. [8]	56 aesthetic features such as light, colorfulness, saturation, hue, rule of thirds, familiarity measure, texture, size, aspect ratio, region composition, low depth of field indicators, and shape convexity.
	Ke et al. [9]	6 aesthetic features such as spatial distribution of edges, color distribution, hue count, blur, contrast, and average brightness.
	Tong et al. [10]	Blurriness, contrast, colorfulness, and saliency.
	Marchesotti et al. [11]	Generic content-based local image signatures.
**Deep learning features**	Tian et al. [12]	A powerful deep convolutional neural network.
	Kao et al. [13]	A multi-task deep learning framework.
	Lu [14]	A double-column deep convolutional neural network.
	Wang et al. [15]	A multi-scene deep learning model.
	Zhang et al. [16]	A multimodal self-and-collaborative attention network.
	Yang et al. [18]	A color composition and space formation model.
	Celona et al. [19]	A model combining image semantics, artistic style, and composition.
	Pfister et al. [20], Yan et al. [21]	Self-attention and semantic-aware multi-task convolutional neural networks.
	Soydaner et al. [2]	A multi-task CNN for joint learning of aesthetic scores and attributes.
	Li et al. [6]	A multimodal memory network using attribute-guided visual-text interaction.
	Pan et al. [7]	An adversarial attribute- assisted aesthetic assessment model.

In the realm of deep learning, the methods listed tend to emphasize the high-level characteristics captured by deep neural networks. Tian et al. [12] noted that CNNs learn the spatial hierarchies in images. However, the emphasis on spatial location is inconsistent and often unclear. Lu [14] and Wang et al. [15] described deep learning models that are likely to learn spatial relationships; however, the explicit focus on spatial location information was not mentioned. Celona et al. [19] discussed a model that combines image semantics, artistic style, and composition, which suggests a broader understanding of spatial location, as composition involves the arrangement of elements in space. However, the specifics of how spatial information is processed are not detailed.

In addition, recent approaches such as the multi-task CNN framework [2], the Attribute-Assisted Multimodal Memory Network(AAMMN) [6], and the adversarial attribute-guided aesthetic [7] have explored auxiliary cues such as multi-task learning, semantic attributes, and multimodal information to enhance aesthetic prediction. These methods have achieved good results by modeling attribute-level supervision or using attention mechanisms. However, they still rely on convolutional backbones, where the loss of spatial detail due to pooling or flattened representations may limit the model’s ability to retain spatial relationships critical to image composition.

In conclusion, although some methods may capture spatial information via CNN structures or image analysis, many do not provide explicit and comprehensive details of the spatial location information. To overcome these shortcomings, this study proposes a novel approach utilizing CapsNet.

**Fig 2:**
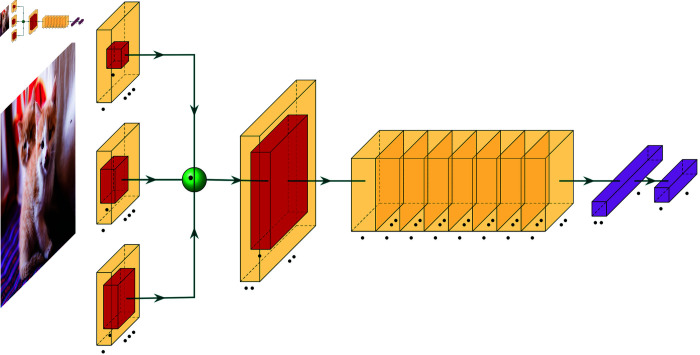
The structure of the DCCN model.

To incorporate the spatial information of images, this study proposes a deep convolutional capsule network as the basic architecture, which integrates a CapsNet and an inception module. The structure of the DCCN model is shown in Fig 2.

The DCCN model consists of four layers: an initial convolutional layer, a primary capsule layer, a digital capsule layer, and an output layer. The initial convolutional layer performs convolutional operations consistent with a conventional neural network, which can extract image features and reduce the number of parameters. The primary capsule layer further performs the convolution operations. The result of the convolution operation is converted into the data format required by the digital capsule layer. The digital capsule layer employs a dynamic routing mechanism to train the network. Finally, the output layer generates the recognition results.

### Initial convolutional layer

Since the introduction of AlexNet in 2012, the trend of neural network architecture has inclined to increase the depth of the network [32]. However, it has been observed that beyond a certain threshold, more layers can lead to a decrease in accuracy. Moreover, a network with a large number of parameters requires numerous hardware resources for training [33,34]. To address this limitation, Google proposed GoogLeNet [35], which offers an alternative to the traditional neural network structures. GoogLeNet primarily utilizes inception modules [36], which combine different convolutional kernels and merge the results into a specific dimension. There are five versions of inception: inception-v1, inception-v2, inception-v3, inception-v4, and inception-resnet.

**Fig 3:**
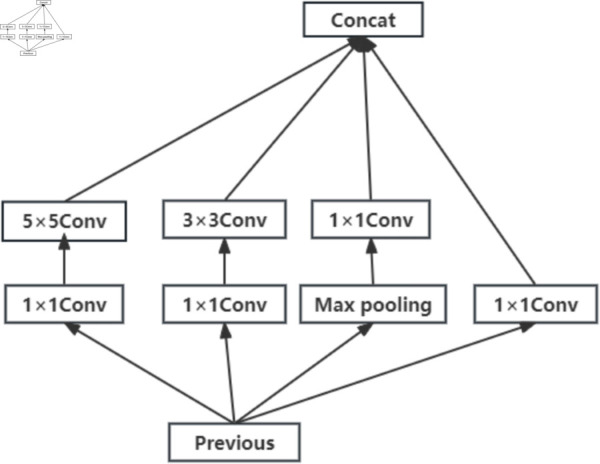
The structure of Inception-v1.

The inception-v1 module includes 1 × 1, 3 × 3, and 5 × 5 convolution kernel channels and pooling layer channels [37]. This module can extract features from three different fields of view by employing convolution kernels with three different sizes. The structure of Inception-v1 is shown in Fig 3.

**Fig 4:**
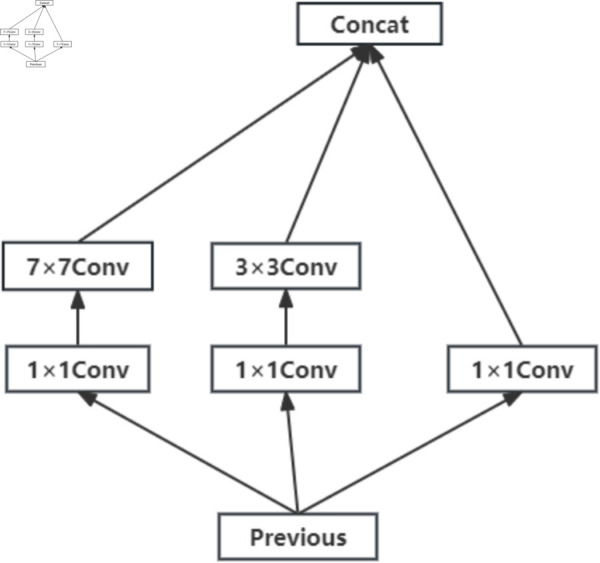
The structure of the improved Inception-v1 module.

The CapsNet proposed by Hinton has only one convolutional layer in the initial convolution stage, and this convolutional layer contains only a single convolution kernel [28]. However, if only one convolutional kernel is used to extract aesthetic features, the obtained features may not be comprehensive, which can significantly affect the assessment accuracy. To address this limitation, in this paper, the Inception-v1 module is used to replace the single convolution kernel [38] in the initial convolutional layer of CapsNet. However, the direct incorporation of the Inception-v1 module in the CapsNet has two limitations. First, the maximum pooling operation within the Inception-v1 module leads to a loss of spatial information in the image. Second, the 1 × 1, 3 × 3 and 5 × 5 convolutional kernels offer limited fields of view, which are inadequate for effectively extracting features from larger fields of view. To capture significant image aesthetic features better, in this paper, two improvements of the Inception-v1 module are presented in the initial convolutional layer. First, to preserve spatial information, the pooling channels in the Inception-v1 module are removed. Second, to extract aesthetic features across both small and large fields of view, the 5 × 5 convolutional kernel in Inception-v1 is replaced with a larger 7 × 7 convolutional kernel. The improved Inception-v1 module is shown in Fig 4.

### Primary capsule layer

To reduce the number of parameters, a 9 × 9 convolutional kernel is utilized in the primary capsule layer to convolve the fused features from the initial convolutional layer further. This produces a feature map of *h* × *w* × 64, where *h* and *w* represent the height and width of the feature map, respectively. To comply with the data format required by the digital capsule layer [39], the matrix of this feature map is rearranged into a data format of r × 8, where the parameter r is equal to hei × wid × 8.

### Digital capsule layer

The vectors outputted from the primary capsule layer are fed into the digital capsule layer. The dynamic routing process is as follows.

1. Input vectors *u*_*r*_ are multiplied by the initialization matrices *w*_*ir*_ to generate shallow capsules *u*_*ir*_, as shown in (1).
  $$u_{ir}=u_{r}\cdot w_{ir}\;i=1,2,3;\;r=1,2,\ldots ,\text{hei}\times \text{wid}\times 8$$ (1)
  where i represents the number of repetitions and r denotes the number of vectors involved.
2. The matrices *b*_*ir*_ are initialized and subjected to the softmax operation. The softmax operation generates the result *c*_*ir*_, as shown in (2). The shallow capsules *u*_*ir*_ are first multiplied by *c*_*ir*_ and then are summed to obtain the deep capsule *s*_*i*_, as shown in (3).
  $$c_{ir}=\frac{\exp(b_{ir})}{\sum_{k}\exp(b_{ik})}\;i=1,2,3;\;r=1,2,\ldots ,\text{hei}\times \text{wid}\times 8$$ (2)
  $$s_{i}=\sum_{r}c_{ir}\cdot u_{ir}\;i=1,2,3;\;r=1,2,\ldots ,\text{hei}\times \text{wid}\times 8$$ (3)
3. A squashing function is performed on the deep capsule *s*_*i*_ to generate the recognition result $v_{i}$. The squashing function is shown in (4).
  $$v_{i}=\frac{\|s_{i}{\|}^{2}}{1+\|s_{i}\|}\cdot \frac{s_{i}}{\left\|s_{i}\right\|}\;i=1,2,3$$ (4)
4. The obtained vector $v_{i}$ is first multiplied by the shallow capsules *u*_*ir*_ and then added to *b*_*ir*_. The updated *b*_*ir*_ is obtained, as shown in (5).
  $$b_{ir}=b_{ir}+v_{i}\cdot u_{ir}$$ (5)
  The above steps (1), (2), (3), and (4) are repeated three times.

### Loss function

The input *x* is fed into the DCCN model to generate the predicted aesthetic score $\hat{y}$, as shown in (6).

$$\hat{y}=f(x;w_{ir})$$ (6)

where *f* denotes the proposed DCCN model, and *w*_*ir*_ represents the parameters that must be initialized. To reduce the loss between the predicted and ground-truth aesthetic scores of image *x*, the boundary loss is used as the loss function, which takes the form of (7).

$$\text{Loss}(y,\hat{y})=y\max(0,m^{+}-\hat{y}{)}^{2}+\lambda (1-y)\max(0,\hat{y}-m^{-}{)}^{2}$$ (7)

where y denotes the ground-truth aesthetic score, *m*^ + ^ represents the upper boundary with a value of 0.9, *m*^−^ stands for the lower boundary with a value of 0.1, and parameter *λ* is set to 0.5, which indicates that the loss of positive samples is twice as important as the loss of negative samples.

The gradient descent method is used to update the matrix *w*_*ir*_, as shown in (8).

$$g_{wir}=\nabla_{wir}\text{Loss}(y,\hat{y})$$ (8)

This study used the Adam optimizer to update *w*_*ir*_, as is shown in (9).

$$\text{Adam(Loss}(y,\hat{y}),w_{ir}):\;w_{ir}^{\prime }\leftarrow w_{ir}-\alpha \sum_{j=1}^{S}\frac{m_{wir}(j)}{\sqrt{v_{wir}(j)+\varepsilon }}$$ (9)

where *ε* denotes a small constant with a value of 1e-5, *α* represents the inner learning rate, and the terms *m*_*wir*_(*j*) and $v_{wir}(j)$ stand for the first and second raw moments of the gradients, defined as (10) and (11), respectively.

$$m_{wir}(j)=\mu_{1}m_{wir}(j-1)+(1-\mu_{1})g_{wir}(j)$$ (10)

$$v_{wir}(j)=\mu_{2}v_{wir}(j-1)+(1-\mu_{2})g_{wir}^{2}(j)$$ (11)

where the values of *m*_*wir*_(0) and $v_{wir}(0)$ are set to zero; $\mu_{1}$ and $\mu_{2}$ denotes the exponential decay rates of *m*_*wir*_(*j*) and $v_{wir}(j)$; *g*_*wir*_(*j*) represents the updated gradients in *j*-th step and *j* ranges from 1 to *S*.

The process of the proposed DCCN model can be roughly described as Algorithm 1.

**Algorithm 1. Algorithm for proposed approach.**



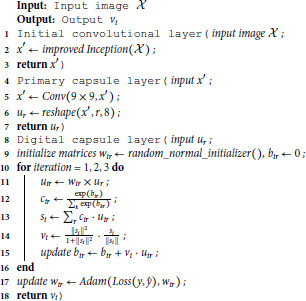



### Process summary

In summary, the complete experimental procedure is as follows: In the data preprocessing stage, we resized the images from the CUHK-PQ and AVA datasets to 112 × 112 and normalized the pixel values to the range of [0, 1]. The core architecture of the DCCN model includes the input layer, the improved inception module, and the capsule network. The inception module is responsible for extracting multi-scale aesthetic features from the images, while the dynamic routing mechanism of the capsule network helps retain the spatial hierarchy, further enhancing the modeling of spatial information. During training, we employed an appropriate loss function and used the Adam optimizer for parameter optimization. In subsequent experiments, we validated the model’s effectiveness using evaluation metrics such as classification accuracy, PLCC, and SROCC.

## Experimental results

In this section, we first outline the experimental configuration, datasets utilized, and evaluation metrics. Furthermore, experiments are conducted on the selection of inception convolution kernel sizes. Then, a discussion of the differing performances of the DCCN model on the CUHK-PQ and AVA datasets is presented. To conclude, this paper presents two sets of ablation studies that confirm the efficacy of the enhanced inception module and boundary loss function.

### Experimental configuration

The experimental configurations are listed in Table 2. The experiments are conducted using Python 3.7.0 with TensorFlow-GPU 2.6.0 as the programming environment. An Intel Core i7-13700 CPU with 64.0 GB of RAM and an NVIDIA RTX 4090 GPU with 24 GB of memory are utilized. The size of the input images is set as 112 × 112 × 3. The training and validation sets are split in an 8:1 ratio, the batch size is set to 128, and the learning rate to 1e-8. The proposed DCCN model is trained for 100 epochs, with each epoch taking approximately 70-80 seconds.

**Table 2:** Experimental configuration and the average speed on a single GPU with a batch size of 128.

Experimental details	
**#Params**	**5.83M**
**Compiling environment**	Tensorflow2.0
**CPU**	Intel(R) Core(TM) i7-13700
**GPU**	NVIDIA RTX 4090
**Time per epoch**	72s
**Time per image**	2.039ms/img

### Databases

**Fig 5:**
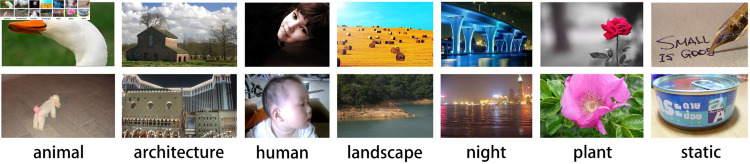
Examples from the CUHK-PQ dataset. The images in the top row have high aesthetic quality, and the images in the bottom row have low aesthetic quality.

The CUHK-PQ [40] dataset is an image aesthetics dataset published by the Chinese University of Hong Kong that includes 17,690 images. Each image was labeled with an aesthetic quality rating of either high or low, as well as semantic annotations. Semantic annotations include categories such as animals, architecture, humans, landscape, night, plants, and static [41]. Fig 5 presents images with different semantic annotations from the CUHK-PQ dataset.

**Fig 6:**
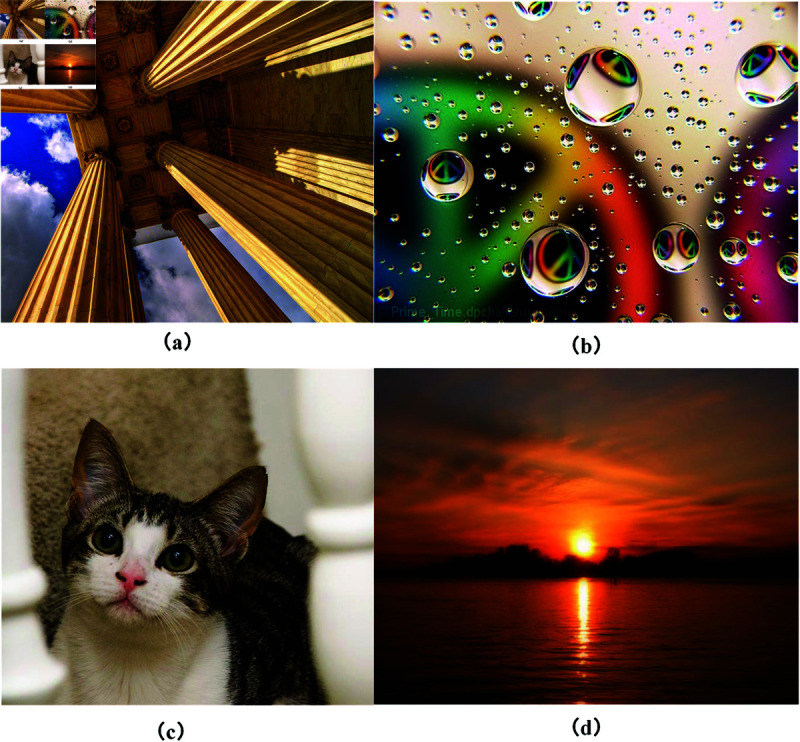
Images of the AVA dataset.

The AVA dataset [42] utilized in the experiments is a large dataset for image aesthetic assessment, containing 250,000 images. Each image in the AVA dataset is annotated using aesthetic scores, semantic labels, and style labels. Aesthetic scores range from 1 to 10 and are provided by different individuals [43]. This dataset includes 66 semantic labels [44], such as animals, history, and music, and each image contains 0-2 semantic annotations. Additionally, there are 14 style labels, including complementary_colors, duotones, HDR, image_grain, light_on_white, long_exposure, macro, motion_blur, negative_image, rule_of_thirds, shallow_DOF, silhouettes, soft_focus, and vanishing_point. Fig 6 presents images from the AVA dataset. Table 3 lists the annotations in Fig 6(a). In the AVA dataset, a score of 5 is commonly considered the threshold distinguishing high and low aesthetic quality images. The score distribution for Fig 6(a) shows that a large number of raters (58 individuals) assigned a score of 7, indicating strong consensus among annotators on the image’s high aesthetic quality. This concentrated distribution in the higher score range reflects the image’s prominent aesthetic appeal and its capacity to attract viewer appreciation.

**Table 3:** AVA example labels.

Scores	The number of evaluators
1	1
2	2
3	6
4	6
5	17
6	50
7	58
8	29
9	16
10	14

### Evaluation indices

The accuracy of an image aesthetics assessment method refers to the capacity of the evaluation model to classify images correctly based on their aesthetic quality. It serves as the most direct index of performance and is quantified by the ratio of the number of images accurately classified by the model to the total number of images. Specifically, in the binary classification task of image aesthetics evaluation, accuracy pertains to the proportion of images that the model precisely differentiates as high- or low- quality. The formula for calculating the accuracy is shown in (12).

$$\text{Accuracy}=\frac{\text{TP}+\text{TN}}{\text{P}+\text{N}}$$ (12)

where *TP* represents the number of high-quality images that are correctly identified as such, *TN* denotes the number of low-quality images that are accurately recognized as low quality,*P* stands for to the total number of positive samples, and *N* is the total number of negative samples.

To measure the strength of the relationship between predicted scores from an image aesthetics assessment method and human subjective scores, this study employs the Pearson linear correlation coefficient (PLCC) and Spearman rank order correlation coefficient (SROCC) as evaluation indices. PLCC and SROCC range from -1 to 1. A PLCC or SROCC value of 1 indicates a perfect positive correlation, meaning the predicted scores are completely proportional to human subjective scores; a value of -1 signifies a perfect negative correlation, meaning the predicted scores are inversely proportional to human subjective scores; and a value of 0 indicates no relationship.

Typically, PLCC measures the strength of the linear relationship between two variables, while SROCC assesses the monotonic relationship regardless of linearity. PLCC is sensitive to outliers because it directly involves numerical values of the raw data. By contrast, SROCC is less sensitive to outliers, as it focuses solely on the rank order of the data.

### Size selection of convolutional kernel in the inception module

The proposed DCCN model is tested on the aforementioned datasets for the training of binary classification. The binary assessment rule is defined in (13).

$$\text{rank}=\left\{ \begin{array}{cc} 0, & \text{if }1\leq \text{score}<5\\ 1, & \text{if }5<\text{score}\leq 10 \end{array}\right.$$ (13)

where *rank* denotes the aesthetic assessment level, *score* represents the mean aesthetic score of an image. The value of *rank* is 0 or 1, which indicates low or high aesthetic quality level.

**Fig 7:**
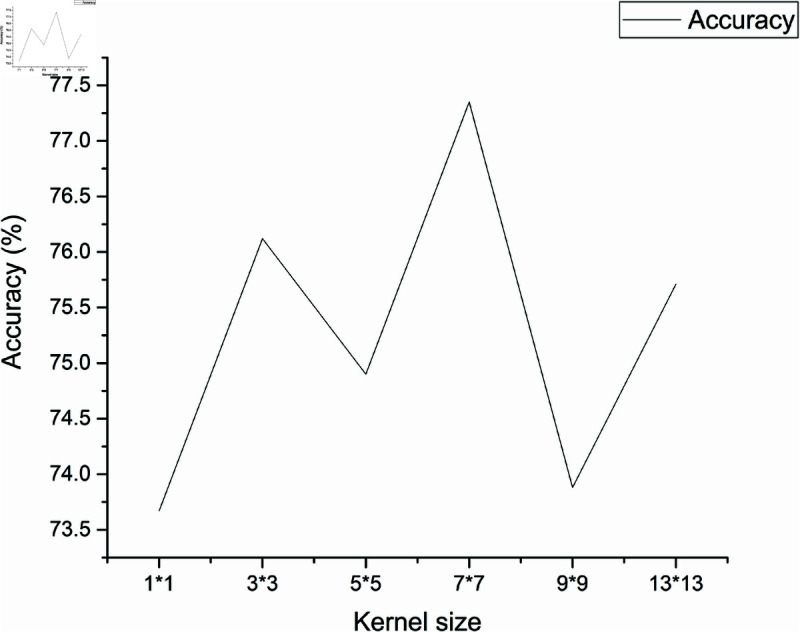
Accuracy of the DCCN which utilizes different convolutional kernels.

In the DCCN model, only the size of the large-field convolution kernel in the initial convolutional layer is modified, whereas the rest of the model remains unchanged. The accuracy results obtained for the AVA dataset are presented in Table 4 and Fig 7.

**Table 4:** Accuracy of the DCCN which utilizes convolutional kernels of different sizes.

Convolution kernel size	Accuracy
1×1	73.67%
3×3	76.12%
5×5	74.90%
7×7	77.35%
9×9	73.88%
13×13	75.71%

In Table 4 and Fig 7, it can be observed that the 7 × 7 convolutional kernel achieves the highest accuracy (77.35%). Hence, 7 × 7 is used as the size of the convolutional kernel with a large field of view in the proposed DCCN model.

We consider that this kernel size offers a good balance between local detail and global context. Smaller kernels (e.g., 1 × 1 or 3 × 3) are limited in capturing high-level aesthetic structures, such as overall composition and balance, while excessively large kernels (e.g., 9 × 9 or 13 × 13) may dilute spatial precision and introduce noise. The 7 × 7 kernel is sufficiently large to capture mid- to high-level aesthetic cues like symmetry, color layout, and saliency regions, while still retaining important spatial details. Therefore, it leads to better feature representation and contributes to improved model performance.

### Peformance of the DCCN model

The accuracy of the proposed DCCN model on both the CUHK-PQ and AVA datasets is shown in Table 5. To reduce the number of parameters, the input image is resized to 112 × 112.

**Table 5:** The performance of the DCCN model on the CUHK-PQ and AVA.

Dataset	Accuracy	PLCC	SROCC
CUHK-PQ	94.79%	-	-
AVA	77.35%	0.8408	0.7394

From Table 5, it can be observed that the DCCN model achieves the best performance with an accuracy of 94.79% on the CUHK-PQ dataset. The second-best performance is obtained on the AVA dataset, with an accuracy of 77.35%. It also attains the PLCC of 0.8408 and the SROCC of 0.7394 for predicting the distribution of aesthetic scores.

The performance gap between the two datasets can be attributed to their inherent differences. CUHK-PQ is a relatively clean and balanced dataset with binary labels (high or low quality), which simplifies the classification task. In contrast, AVA is more complex and challenging—it contains subjective annotations with aesthetic scores ranging from 1 to 10, introducing greater variability and label noise. Additionally, the AVA dataset includes diverse image content and styles, which increases the difficulty of capturing consistent aesthetic patterns. These factors contribute to the lower accuracy observed on AVA compared to CUHK-PQ.

A comparison of the different methods for the CUHK-PQ and AVA datasets is presented in Table 6. While the DCCN model exhibits competitive performance across most evaluation metrics, we note that some prior methods report slightly higher results on certain indicators, particularly on the AVA dataset. This variation may be attributed to differences in model design objectives and dataset characteristics. For instance, models incorporating additional semantic supervision or handcrafted priors may gain advantages in capturing subtle aesthetic cues embedded in the subjective and diverse AVA annotations. In contrast, our model emphasizes structural compactness and spatial hierarchy preservation, aiming for a lightweight yet robust solution that generalizes well across tasks and platforms.

**Table 6:** Comparison of different methods.

Methods	Classification Accuracy		Distribution		#Params
	CUHK-PQ	AVA	PLCC	SROCC	
Su et al. [45]	92.06%	-	-	-	-
Marchesotti et al. [11]	89.90%	-	-	-	-
Zhang et al. [46]	90.31%	-	-	-	-
Tian et al. [12]	91.94%	-	-	-	-
Wang et al. [15]	92.59%	-	-	-	-
AVA manual features [42]	-	68.00%	-	-	-
Kong et al. [47]	-	77.33%	-	-	-
Kao et al. [48]	-	74.51%	0.5214	-	-
GloRe [49]	-	83.46%	0.6480	0.663	25.8M
HLA-GCN [50]	-	84.1%	0.656	0.678	38.2M
Munan et al. [33]	-	80.9%	0.725	0.724	-
IAACS [18]	-	86.29%	0.8003	0.8286	-
Our(DCCN)	94.79%	77.35%	0.8408	0.7394	5.83M

Nevertheless, the DCCN model exhibited an impressive accuracy of 94.79% on the CUHK-PQ dataset, surpassing the Su method [45], Marchesotti method [11], Zhang method [46], Tian method [12], and Wang method [15] by 2.73%, 2.85%, 4.89%, 4.48%, and 2.20%, respectively. This indicates that the DCCN is especially effective in handling binary classification tasks like CUHK-PQ.

The comparative analysis of different methods based on PLCC and SROCC indices, as shown in Table 6, reveals that the DCCN achieves an SROCC of 0.7394, which is 0.0892 lower than the highest score of 0.8286. Nevertheless, it demonstrates superior performance in terms of PLCC, reaching 0.8408. This suggests that the model can better capture the distribution trend of aesthetic scores, even if the ranking is not perfectly aligned.

Moreover, the DCCN model has the fewest parameters (5.83 million) among all methods, whereas the GloRe model [47] has 25.8 million and the HLA-GCN [50] has 38.2 million parameters. A model with fewer parameters is generally less complex, leading to faster inference and lower memory usage. This makes DCCN especially suitable for real-time applications and deployment on resource-constrained or lightweight devices, enhancing its practical value.

Data analysis reveals that the proposed DCCN method achieves higher accuracy on the CUHK-PQ dataset. It also exhibits a higher PLCC value, indicating a strong correlation between the model’s predictions and the actual values. Moreover, compared with other methods, the number of parameters from the DCCN has a smaller value, which results in faster execution times.

The enhanced performance of the DCCN model can be attributed to the strengths of capsule networks, particularly their ability to preserve spatial hierarchies in images. Unlike traditional CNNs that rely heavily on pooling operations, which can cause the loss of important spatial information, the dynamic routing mechanism in capsule networks ensures that the spatial relationships between image features are maintained. This makes capsule networks more suitable for tasks that require spatial understanding, such as image aesthetics assessment, where the positioning and arrangement of visual elements play a crucial role. Additionally, the use of capsules allows the model to capture more complex patterns and hierarchies, improving its ability to recognize fine-grained aesthetic features and making it less sensitive to small variations in image style or composition. The combination of these advantages contributes significantly to the DCCN model’s ability to effectively assess image aesthetics.

To verify the statistical significance between DCCN and recent methods, a Krus-kal-Wallis H test was performed on GloRe, HLA-GCN, Munan, IAACS, and DCCN, considering accuracy, PLCC, SROCC, and the number of parameters as the dependent measures. Statistical analysis indicated that a larger H-statistic typically signifies substantial differences between groups. A p-value less than 0.05 warrants rejection of the null hypothesis. The findings of this study yielded an H value of 13.81 and a p-value of 0.0032, significantly below the threshold of 0.05, providing robust statistical evidence to reject the null hypothesis and affirm the existence of significant disparities.

Table 7 compares the performance of 15 image aesthetic assessment models on the AVA dataset, with all models pre-trained on ImageNet-1K. The table categorizes models into three types: CNN-based, Transformer-based, and the Capsule Network-based model proposed in this study. Each model is evaluated using PLCC, SROCC, and a calculated Ratio metric. In addition, we introduce a derived metric named Ratio, defined as SROCC divided by classification accuracy. This metric reflects the model’s ranking ability normalized against classification performance, offering a more comprehensive view of the model’s generalization ability across both tasks.

**Table 7:** Comparing 16 models on AVA, All models were pre-trained on ImageNet-1K.

Model Type	Metric (AVA)	Input Size	Params	Epoch	PLCC↑	SROCC↑	Ratio↑
CNN-based models	**NIMA [4]**	224	56M	40+	0.636	0.612	0.751
	**ALamp [51]**	224	99M	50+	0.671	0.666	0.807
	**AFDC [52]**	230	45M	-	0.671	0.649	0.779
	**HGCN [53]**	224	44M	60+	0.687	0.665	0.786
	**MLSP [54]**	629	24M	40+	0.757	0.756	0.925
	**TANet [55]**	224	40M	110+	0.765	0.758	0.940
	**ResNext [56]**	512	43M	-	0.781	0.780	0.940
	**POC [57]**	640	1853M	-	0.795	**0.794**	**0.955**
Transformer-based models	**PSViT [58]**	B224	21M	70+	0.645	0.701	0.847
	**DPT [59]**	L224	61M	100+	0.720	0.694	0.874
	**Swin [60]**	B224	87M	70+	0.737	0.736	0.901
	**MUSIQ [61]**	512	87M	90+	0.738	0.726	0.891
	**DAT [62]**	B224	87M	60+	0.739	0.738	0.909
	**MaxViT [63]**	512	31M	300+	0.745	0.759	0.877
Capsule-based models	**Ours**	112	**5.83M**	100+	**0.840**	0.739	0.955

The DCCN model, which is the focus of this study, belongs to the Capsule-based category. It achieves a PLCC of 0.840 and an SROCC of 0.739 on the AVA dataset, surpassing most CNN-based and Transformer-based models in terms of PLCC. Despite having only 5.83M parameters, DCCN demonstrates a highly favorable balance between predictive performance and computational efficiency. Specifically, the Ratio value reaches 0.955, indicating that the model delivers exceptional performance relative to its parameter size, even outperforming many models with substantially larger complexities.

In conclusion, the DCCN model excels in performance, particularly in terms of the PLCC metric, while maintaining a much smaller model size compared to other state-of-the-art methods. This result highlights the efficiency of the capsule network in capturing spatial features, enhancing aesthetic quality evaluation while minimizing computational cost. The DCCN’s competitive performance, paired with its small parameter size, makes it a highly efficient model for real-time applications, especially when considering resource-constrained environments.

### Ablation study

In this subsection, we explore the efficacy of the DCCN model using four sets of ablation experiments. The first set aims to assess the impact of the improved inception module on the DCCN model. The second set evaluates the effects of different loss functions on the performance of the DCCN model. The third set investigates the influence of varying the number of dynamic routing iterations in the capsule network, testing the model’s performance with 2, 3, 4, and 5 iterations. Finally, the fourth set explores the impact of modifying the number of capsules in the DigitCaps layer, analyzing how different capsule configurations affect the model’s overall performance.

**Fig 8:**
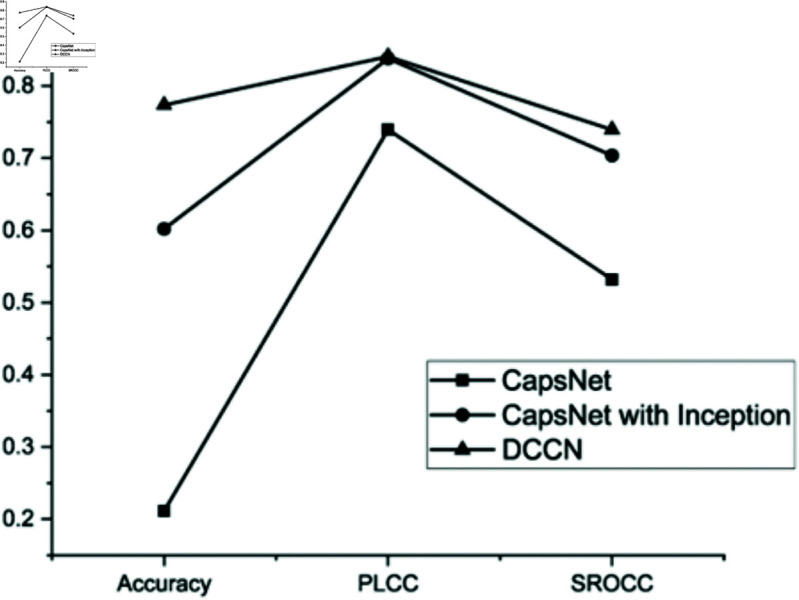
Comparative chart of the first set of ablation study results.

The first set of ablation experiments is conducted to verify the effectiveness of the improved inception module, which comprises three trials. Initially, a capsule network was selected as the backbone of our architecture because it can have better capability in capturing image features and simultaneously preserving spatial positional information. Subsequently, to extract more effective aesthetic features, an inception module was incorporated into the capsule network. Finally, an enhancement of the inception module is proposed to prevent the loss of spatial information that may result from max-pooling operations. The results of these experiments are presented in Table 8 and Fig 8.

**Table 8:** Results of the first set of ablation studies.

Methods	Accuracy in AVA	PLCC	SROCC
The capsule	21.14%	0.7395	0.5321
The capsule with the inception module	60.21%	0.838	0.7037
The DCCN	77.35%	0.8408	0.7394

It is evident that the DCCN model outperforms both the CapsNet and CapsNet with the inception module in terms of performance. This indicates that the DCCN model is capable of effectively capturing the aesthetic features of images while maintaining stable performance. The effectiveness of the improved inception module has also been validated through our experiments.

**Fig 9:**
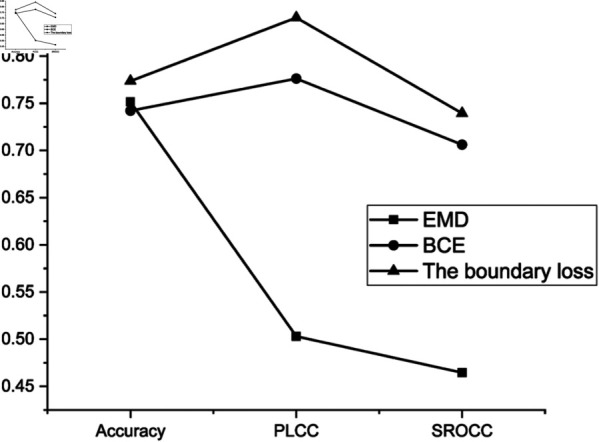
Comparative chart of the second set of ablation study results.

The second set of ablation experiments is conducted to verify the effectiveness of the boundary loss, which includes three distinct trials, and the results of experiments are presented in Table 9 and Fig 9. Under the precondition that the DCCN model structure remains unaltered, this study compares its performance using EMD loss, cross-entropy loss, and boundary loss. The corresponding experimental results are shown in Fig 8(b). For the true values P and predicted values $\hat{P}$, the EMD loss is computed, as shown in (14).

**Table 9:** Results of the second set of ablation studies.

Loss	Accuracy in AVA	PLCC	SROCC
EMD	75.15%	50.30%	46.46%
BCE	74.21%	77.62%	70.62%
The boundary loss	77.35%	84.08%	73.94%

$$\text{EMD}(P,\hat{P})=\sum_{i=1}^{n}\left|\sum_{k=1}^{i}p_{k}-\sum_{k=1}^{i}{\hat{p}}_{k}\right|$$ (14)

where *p*_*k*_ and ${\hat{P}}_{k}$ denotes the probability masses of P and $\hat{P}$ the k-th point, respectively. The cross-entropy loss is computed, as shown in (15).

$$\text{BCE}(P,\hat{P})=-P\log(\hat{P})-(1-P)\log(1-\log\hat{P})$$ (15)

The third set of ablation experiments investigates the influence of the number of dynamic routing iterations in the capsule network. This experiment tests the DCCN model with different numbers of routing iterations: 2, 3, 4, and 5. The aim is to analyze how varying the number of dynamic routing iterations impacts the model’s performance in terms of classification accuracy, PLCC and SROCC.

The results of these experiments are presented in Table 10. As shown in the results, the DCCN model achieves its best performance with 3 iterations of dynamic routing, which leads to an optimal balance between spatial representation and computational efficiency. Increasing the number of routing iterations beyond 3 does not significantly improve performance but increases the computational cost. This demonstrates that the DCCN model benefits from an optimal number of routing iterations, where 3 iterations provide the best trade-off between model accuracy and computational efficiency.

**Table 10:** Results of the third set of ablation studies.

Trial	Accuracy in AVA	PLCC	SROCC
2	74.30%	0.8186	0.6902
3	77.35%	0.8408	0.7394
4	75.90%	0.8323	0.7173
5	74.75%	0.8203	0.7058

The fourth set of ablation experiments investigates the effect of different structural configurations of the DigitCaps layer while keeping the total number of capsules constant (64). Specifically, we evaluate three capsule arrangements: 32 × 2, 16 × 4, and 8 × 8. As presented in Table 11, the 8 × 8 structure used in the DCCN model achieves the best performance across all metrics, indicating that a more balanced and square-shaped capsule layout facilitates more stable dynamic routing and more effective spatial representation.

**Table 11:** Results of the fourth set of ablation studies (Different Capsule Configurations in DigitCaps Layer).

Capsule Configuration	Accuracy (AVA)	PLCC	SROCC
32 × 2	74.30%	0.8186	0.6902
16 × 4	75.90%	0.8323	0.7173
8 × 8 (DCCN)	**77.35%**	**0.8408**	**0.7394**

From Table 8 and Fig 9, it can be observed that within the DCCN model, the accuracy attained with the boundary loss reaches 77.35%, PLCC reaches 84.08%, and SROCC reaches 73.94%. These indices surpass those achieved using EMD and cross-entropy losses. Consequently, this study posits that employing boundary loss in the DCCN model is more effective than utilizing EMD or cross-entropy loss. From the results presented in Tables 8 to 11, it is evident that each component of the DCCN model contributes significantly to its overall performance. The boundary loss demonstrates superior effectiveness, with the DCCN achieving an accuracy of 77.35%, a PLCC of 84.08%, and an SROCC of 73.94%, outperforming both the EMD and cross-entropy losses. Similarly, the improved inception module markedly enhances feature extraction capability, as shown by the substantial performance gap between the baseline capsule network and the final DCCN architecture. Furthermore, experiments modifying the number of routing iterations and the configuration of the DigitCaps layer also confirm that the default settings used in the DCCN — three routing iterations and an 8 × 8 DigitCaps layout — achieve the best balance between accuracy and stability. These ablation results comprehensively validate the architectural and training choices made in the design of the DCCN model.

### Pedictions of model

Fourteen images with different semantic annotations from the CUHK-PQ and AVA datasets are randomly selected for prediction.

**Fig 10:**
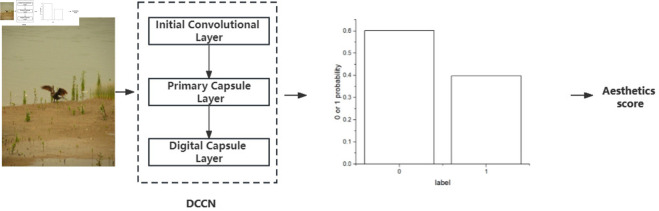
The assessment process.

**Fig 11:**
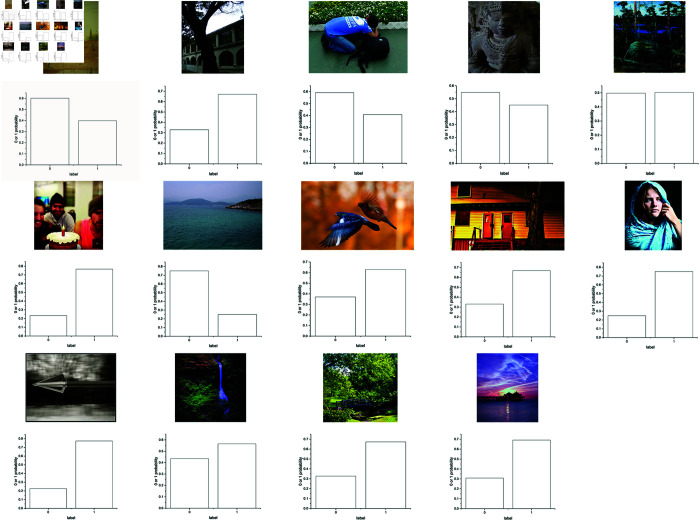
14 images selected from CUHK-PQ and AVA and their corresponding probability values obtained from the DCCN model. (a)–(g) are low aesthetic quality images, and (h)–(n) are high aesthetic quality images.

The assessment process is illustrated in Fig 10. The ratio of high to low aesthetic quality images is 1:1, which includes seven images of high aesthetic quality and seven of low aesthetic quality. Fig 11 illustrates these selected images and their probabilities obtained from the proposed DCCN model. The original labels and predictions of these fourteen images are provided in Table 12, where 1 and 0 denote respectively high aesthetic quality and low aesthetic quality.

**Table 12:** Original labels and prediction for 14 images.

Images	Original Label	Predictive Value	True or False
Image 11(a)	0	0	T
Image 11(b)	0	1	F
Image 11(c)	0	0	T
Image 11(d)	0	0	T
Image 11(e)	0	1	F
Image 11(f)	0	1	F
Image 11(g)	0	1	F
Image 11(h)	1	1	T
Image 11(i)	1	1	T
Image 11(j)	1	1	T
Image 11(k)	1	1	T
Image 11(l)	1	1	T
Image 11(m)	1	1	T
Image 11(n)	1	1	T

From the experimental results in Table 12, it can be seen that ten images are correctly classified and four images are misclassified. Thus, the proposed DCCN model achieves a prediction accuracy of 71.43%, as listed in Table 12. This further confirms the effectiveness of the DCCN model.

**Fig 12:**
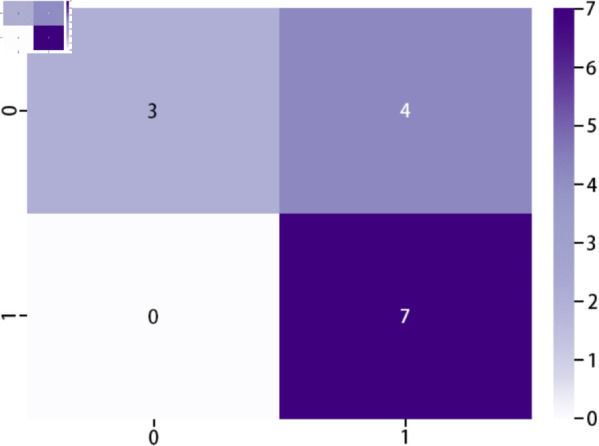
Confusion matrix heatmap.

The confusion matrix heatmap of these fourteen images is shown in Fig 12. From this heatmap, it can be observed that the DCCN model performs better in predicting images with high aesthetic quality, although its performance is weaker for images with low aesthetic quality.

## Conclusion and future work

This study proposes a novel method for image aesthetics assessment based on CapsNet, designated as the DCCN model. Within the DCCN framework, an improved inception module is introduced to enhance the extraction of aesthetic features. To the best of our knowledge, this is the first application of CapsNet in the image aesthetics assessment domain. The effectiveness of the DCCN model is validated through binary classification and distribution prediction tasks. In the binary classification task, the model achieves an accuracy of 94.79% on CUHK-PQ and 77.35% on AVA. For aesthetic score distribution prediction on the AVA dataset, the DCCN attains a PLCC of 0.8408 and an SROCC of 0.7394, demonstrating its robust performance.

Although the proposed DCCN model exhibits notable performance, especially on the CUHK-PQ dataset, several limitations warrant further investigation. One concern is the model’s sensitivity to variations in image styles, such as vintage filters or grayscale effects, which may hinder its generalization when encountering stylistic distributions not represented in the training set. Additionally, the reliance on fixed-size inputs (112 × 112) may reduce the model’s adaptability across datasets with diverse image resolutions. Furthermore, the dynamic routing mechanism—despite enhancing spatial representation—imposes a relatively high computational cost during inference, which may limit deployment in real-time or resource-constrained scenarios.

Furthermore, future research should also investigate the impact of dataset bias on the model’s performance. The DCCN model currently shows sensitivity to different dataset distributions, which can lead to performance degradation. To address this issue, domain adaptation techniques could be explored to improve the model’s generalization across various datasets and reduce the impact of dataset-specific bias.

To further enhance the model’s effectiveness, future work can focus on expanding the diversity of the training dataset to include a broader spectrum of compositional styles and aesthetic patterns. Incorporating additional image-level information, such as semantic or emotional attributes, may also contribute to more nuanced aesthetic evaluation. Moreover, exploring advanced capsule-based architectures (e.g., Matrix Capsules) or hybrid frameworks that integrate DCCN with other neural networks may improve both performance and generalization. Addressing the aforementioned limitations could ultimately strengthen the practical applicability and robustness of the DCCN model in real-world aesthetic quality assessment tasks.
